# Cognitive Function in Patients With Complex Regional Pain Syndrome (CRPS)

**DOI:** 10.1002/ejp.70070

**Published:** 2025-07-08

**Authors:** S. Liesto, T. Aho, S. K. Jääskeläinen, M. Hietanen, E. Kalso

**Affiliations:** ^1^ Dept. of Anaesthesiology, Intensive Care and Pain Medicine University of Helsinki and Helsinki University Hospital Helsinki Finland; ^2^ Outpatient Clinic for Persistent Symptom Rehabilitation University of Helsinki and Helsinki University Hospital Helsinki Finland; ^3^ Department of Clinical Neurophysiology University of Turku and Turku University Hospital Turku Finland; ^4^ Neurocenter, Neuropsychology University of Helsinki and Helsinki University Hospital Helsinki Finland

## Abstract

**Background:**

Complex Regional Pain Syndrome (CRPS) is characterised by pain, sensory, vasomotor, sudomotor, motor, and trophic symptoms, with cognitive symptoms also reported. This study aimed to examine the neuropsychological profile of patients with meticulously diagnosed CRPS 1 and 2.

**Methods:**

A neuropsychological examination was conducted in 54 CRPS patients. The test battery included Block Design and Similarities from Wechsler Adult Intelligence‐IV, word list learning and delayed recall, and Digit Span from Wechsler Memory Scale‐III, Trail Making Test (TMT), verbal and drawing fluency, Brixton, Manikin and Bourdon–Wiersma tests. Patients performing below −0.67 SD of the performance of a healthy Finnish population in ≥ 3 tests were classified as having cognitive decline. Questionnaires included Insomnia Severity Index, Beck Depression Inventory‐II, CRPS Severity Scale, Disabilities of the Arm, Shoulder and Hand, Pain Catastrophizing Scale, Resilience Scale‐14, Brief Pain Inventory, Insomnia Severity Index and State Trait Anxiety Inventory. Patients were asked about subjective cognitive difficulties.

**Results:**

A subgroup comprising 30% of the CRPS patients showed cognitive decline. Higher pain catastrophising was associated with poorer test performance in TMT Part A. There were no differences between left and right sides on the visual attention test. Subjective cognitive difficulties were not associated with objective cognitive test performance. Cognitive functioning did not differ between CRPS 1 and 2 patients.

**Conclusions:**

This study elucidates how CRPS affects cognitive functioning, important information when tailoring multidisciplinary rehabilitation to CRPS patients. Psychological factors may have a stronger impact on subjective cognitive difficulties than on objective test performances.

**Significance Statement:**

CRPS is an enigmatic syndrome with multifactorial origins, leading to chronic, disabling pain due to progressing neuroplastic alterations in the central nervous system. This study adds comprehensive novel information about cognitive function in CRPS, as, except for lateralised cognitions and body perception, cognitive domains have not previously been extensively studied in CRPS patients. Cognitive decline in 30% of patients indicates that neuropsychological assessment should be included in the diagnostics, and results considered in the rehabilitation of CRPS patients.

**Trial Registration:**

ClinicalTrials.gov identifier: NCT04439669

## 1 Introduction

Complex Regional Pain Syndrome (CRPS) is a debilitating condition that typically emerges following trauma to an extremity. It is characterised by pain, sensory, vasomotor, sudomotor, motor, and trophic symptoms and signs, which significantly impact quality of life. Diagnosis of CRPS type 2, but not type 1, requires a clinically evident neuropathic lesion. The incidence of CRPS is estimated at between 5 and 26 per 100,000 individuals per year (de Mos et al. 2007; Sandroni et al. 2003).

The pathophysiology of CRPS remains unclear, involving an interplay of inflammatory, immunological, and autonomic changes, as well as central and peripheral sensitisation. Initial inflammatory and immunological responses may lead to peripheral and central sensitisation, involving plastic changes in the spinal cord and somatosensory cortex. Cortical reorganisation with reduction in both motor and somatosensory cortices corresponding to the extremity affected by CRPS and abnormal neural activity in brain areas essential for sensorimotor function and pain have been reported (Hotta et al. 2017, 2023; Kirveskari et al. 2010; Swart et al. 2009; Zangrandi et al. 2021).

Neuropsychological changes in CRPS have traditionally been described as ‘neglect‐like’ syndromes, referring to spatial attention bias away from the affected side (Galer et al. 1995). Distorted body representation and deficits in lateralized spatial cognition have been reported in CRPS patients but symptoms appear broader than merely ‘neglect‐like’ symptoms (Halicka et al. 2020). Non‐spatially lateralized cognitive function including domains such as memory, executive function, and generalised attention, have been less studied with mixed results (Halicka et al. 2020). Up to 65% of CRPS patients have been reported to have significant neuropsychological deficits, including dysfunction in executive and memory functions (Libon et al. 2010). Some patients present with rare neuropsychological symptoms such as agnosia or apraxia (Cohen et al. 2013).

Depression is the most often reported psychiatric comorbidity in CRPS patients (Duong et al. 2020), and cognitive deficits frequently accompany depression (Kriesche et al. 2023). Pain catastrophizing refers to an exaggerated negative mental set during actual or anticipated painful experiences (Sullivan et al. 2001). In CRPS patients, it is frequent (Im et al. 2021), and associates with greater disability and pain (Farzad et al. 2022).

Psychological resilience refers to the ability to maintain or recover psychological and physical functioning during or after significant life adversities (Bonanno et al. 2011), helping in coping with chronic pain (Finlay et al. 2021). In CRPS patients, resilience is directly associated with quality of life and pain self‐efficacy, and inversely with anxiety, depressive symptoms, and fatigue (Wertli et al. 2023). Among healthy volunteers, higher resilience and lower pain catastrophising correlated with better short‐term memory performance during experimental pain (Ysidron et al. 2021).

This study aims to examine the neuropsychological profile of CRPS 1 and 2 patients compared with a healthy population to elucidate the subgroup of CRPS patients with cognitive decline. The second aim was to test how pain and other CRPS symptoms, type of CRPS, psychological resilience, and pain catastrophising are associated with cognitive performance among CRPS patients.

## 2 Methods

The study was approved by the Coordinating Ethics Committee of the Helsinki and Uusimaa Hospital District (reference number 45/13/03/01/2013) and registered in ClinicalTrials.gov. All eligible patients gave written informed consent.

### 2.1 Patients

This study is part of a prospective two‐centre study on the efficacy of repetitive Transcranial Magnetic Stimulation (rTMS) in CRPS patients and it reports on data relevant to this subproject, collected at the study baseline. The results of the randomised controlled trial intervention will be reported elsewhere. The inclusion criteria were CRPS 1 or 2 of the upper limb diagnosed according to the Budapest criteria for research (Harden et al. 2010), with duration ≥ 6 months, age ≥ 18 years, mean pain intensity ≥ 5/10 (numerical rating scale (NRS) 0–10: 0 no pain; 10 worst pain), conventional therapies had been tried or continued without significant relief. Only patients who were fluent in the Finnish language were included. Exclusion criteria were other ongoing stimulation therapies (apart from transcutaneous nerve stimulation (TNS)), a psychotic disorder, use of strong opioids, severe major depression, epilepsy, any contraindications for MRI, abuse of alcohol or drugs, and ongoing insurance or other entitlement cases. Screening and clinical assessment of the patients was conducted by medical doctors trained for the research protocol. The screening and clinical assessment took in average 2.5 h including informing the patient about the study protocol. Brain MRI was performed on all participants for navigated rTMS: patients with clinically significant findings were excluded from this study, as were patients with subsequently diagnosed neurological disorders that might affect cognitive function.

Participants were recruited from the Helsinki and Turku University Hospital Pain Clinics, private clinics, and via advertisements on social media in a private CRPS patient group. Of these, 57% were examined and treated at the Helsinki University Hospital and 43% at the Turku University Hospital between October 2017 and October 2021.

### 2.2 Neuropsychological Tests

The neuropsychological tests used in this study are described in Table 1.

**TABLE 1:** Neuropsychological tests used in this study.

Test (selected variable)	Units	Direction of performance	References	Finnish population values−
Reasoning				
Block design (WAIS‐IV)	Points (raw score)	+	Wechsler 2012	Wechsler 2012
Similarities (WAIS‐IV)	Points (raw score)	+	Wechsler 2012	Wechsler 2012
Memory				
Word list (WMS‐III)	*n* of words	+	Wechsler 2008	Wechsler 2008
Word list delayed recall (WMS‐III)	*n* of words	+	Wechsler 2008	Wechsler 2008
Digit span forward (WMS‐III)	Points (raw score)	+	Wechsler 2008	Wechsler 2008
Digit span backward (WMS‐III)	Points (raw score)	+	Wechsler 2008	Wechsler 2008
Attention				
TMB	Seconds	−	Lezak et al. 2012	
TMB‐TMA	Difference in seconds	−	Lezak et al. 2012	Poutiainen et al. 2010
Executive function				
Verbal fluency phonemic	*n* of words	+	Lezak et al. 2012	Kivisaari et al. 2009
Drawing fluency	*n* of correct drawings	+	Jones‐Gotman and Milner 1977	—
Brixton	Seconds	−	Burgess and Shallice 1997	—
Brixton errors	*n* of errors	−	Burgess and Shallice 1997	—
B–W dual task decrement	Percentage decrement in dual task vs. single task	−	Vilkki et al. 1996	—
Processing speed				
TMA	Seconds	−	Lezak et al. 2012	Poutiainen et al. 2010
Spatial orientation				
Manikin	Seconds	−	Carter and Woldstad 1985	—

*Note:* + indicates higher scores representing better performance; − indicates lower scores representing better performance. Drawing fluency fixed four‐line condition modified to be completed in 1 min and number of correct drawings calculated. Only trials 1 to 4 conducted in the B–W test and the dual task decrement calculated from trials 2 and 4.

Abbreviations: Brixton, Brixton spatial anticipation test; B–W, Bourdon–Wiersma dot cancellation test; Manikin, spatial orientation Manikin Test, modified to have 10 stimuli total time to complete task recorded; TM, Trail Making; WAIS‐IV, Wechsler Adult Intelligence Scale, 4th edition; WMS‐III, Wechsler Memory Scale, 3rd edition.

Neuropsychological assessment took on average 1 h to complete and was conducted in a single session. The assessment was conducted by a trained clinical psychologist (S.L. and T.A.). Psychological questionnaires were completed at a separate research session with other clinical assessments and took on average 30 min to fill in. Since patients had upper extremity CRPS, they were only able to use one hand to complete Block Design. In Block Design and TMT, depending on the hand affected by CRPS, some patients completed the task with their nondominant hand. The test manual instructions were applied in the calculation of the scores, and neither adjustments nor bonuses were used. Patients filled out the psychological questionnaires as part of the initial screening and clinical assessment visit, where these questionnaires took approximately 30 min to fill in. In the neuropsychological examination, patients were also asked to give their subjective perspective on the following questions: (1) Has CRPS affected your memory and information processing? (yes/no). (2) How good is your present memory and information processing? (0–10 scale: 0 very bad; 10 very good).

Since age affects cognitive performance, age‐corrected values of test scores are used in clinical practice. Information about average test performance in different age groups in the Finnish population was used to convert test performances into age‐corrected values. Wechsler tests use Intelligence Quotient score 90–110 to represent average performance, which means standard deviation (SD) ±0.67 of population performance (Wechsler 2012). Therefore, we used SD ≥ 0.67 below expected mean to be the clinical cutoff point for the tests in this study. Finnish normative values were available for Wechsler tests, verbal fluency, and Trail Making Test (TMT). In this study, we defined patients performing under the clinical cutoff point (−0.67 SD) on three or more tests as having neurocognitive decline, corresponding to the neuropsychological criteria of Mild Cognitive Impairment in the framework of dementia risk (Alfano et al. 2022). For this classification, we used Similarities, Word list, Word list delayed recall, Digit Span forward, Digit Span backward, and phonemic verbal fluency which do not need motor function to complete the task. Raw scores from test performances were used in linear regression models, with age as a covariate. Multiple factors are known to affect cognitive functioning. These include age (Tucker‐Drob et al. 2019), medication (Allegri et al. 2019), depressive symptoms (Kriesche et al. 2023) and insomnia (Brownlow et al. 2020) which is the reason we controlled these factors in regression models. Anxiety and depression have high comorbidity (Belzer and Schneier 2004) and due to problems with multicollinearity in regression models we decided to include only depression.

### 2.3 Questionnaires

The questionnaires used in this study are presented in Table 2. Patients filled in the Brief Pain Inventory (BPI) Short Form for CRPS pain during the previous week. For Pain Interference, we calculated the subscales Active and Affective, according to the test manual of the BPI (Cleeland and Ryan 1994).

**TABLE 2:** Questionnaires used in this study.

Questionnaire	Acronym	Object	Scale	Higher score indicates	References
CRPS Severity Scale	CSS	Number of CRPS symptoms	[0–16]	Greater CRPS severity	Harden et al. 2017
Beck Depression Inventory‐II	BDI‐II	Depressive symptoms	[0–63]	More depressive symptoms	Beck et al. 1961
Disabilities of the arm, shoulder, and hand	DASH	Region specific function disability of upper extremity	[0–100]	Greater disability	Hudak et al. 1996
Pain Catastrophizing Scale	PCS	Pain catastrophizing	[0–52]	Greater catastrophizing	Sullivan et al. 1995
Resilience Scale‐14	RS‐14	Psychological resilience	[14–98]	Greater resilience	Wagnild and Young 1993
Brief Pain Inventory	BPI	Pain intensity and pain interference	[0–10]	Higher pain intensity or interference	Cleeland and Ryan 1994
Insomnia Severity Index	ISI	Insomnia symptoms	[0–28]	More insomnia symptoms	Morin et al. 2011
State Trait Anxiety Inventory^a^	STAI	State and trait anxiety	[20–80]	More anxiety	Spielberger et al. 1983

^a^ Only state anxiety used in this study.

### 2.4 Statistical Analyses

Questionnaires with missing values amounting to more than 20% of the questionnaire were removed from the analysis. Questionnaires with less than 20% missing values (*n* = 10) were included in the analysis after replacing the missing values with the average score of the participants' responses to the same questionnaire, and the sum score averaged by the number of complete responses. Mean imputation was used because the prevalence of missing values was small and assumed to be random. We used a strict 20% missing value threshold because we used mean imputation instead of more advanced imputation methods. In Disabilities of Arm, Shoulder, and Hand (DASH), only 10% missing values were allowed, according to the test manual (*n* = 0). After replacing missing values, depending on the questionnaire, data were available from 53 to 54 patients. All variables under examination were checked for normality by skewness and kurtosis indicators and the Shapiro–Wilk test for normality. Means and SDs are reported for normally distributed variables. Medians and inter‐quartile ranges (IQR) are reported for non‐normally distributed variables.

Independent samples *t*‐tests were used to compare independent subgroups for single dependent variables (patients who had reported that CRPS had or had not affected their cognition; cognitive decline vs. no cognitive decline; CRPS type 1 vs. type 2). The associations of those categorical groups were tested with chi‐squared tests of independence. Linear regression models were used to test whether clinical CRPS‐related or psychological factors were associated with neuropsychological test scores. These models were controlled for depressive symptoms, insomnia symptoms, age, and medication. Binary logistic regression analyses were used to test whether the type of CRPS (CRPS 1 vs. CRPS 2) was associated with cognitive test performance, these analyses being controlled for depressive symptoms, insomnia symptoms, age, and medication. Related‐samples Wilcoxon signed‐rank tests were used to compare the number of errors in performance between right and left sides in the visual attention (Bourdon–Wiersma) test.

All statistical tests were performed using SPSS Version 25. A *p* < 0.05 was considered significant. When conducting the same statistical test multiple times, Bonferroni corrections were used. As there were 15 neuropsychological tests, the level of significance was set to 0.003. With 10 psychological and clinical parameters used, the level of significance was set to 0.005.

## 3 Results

### 3.1 Descriptive Statistics

**FIGURE 1:**
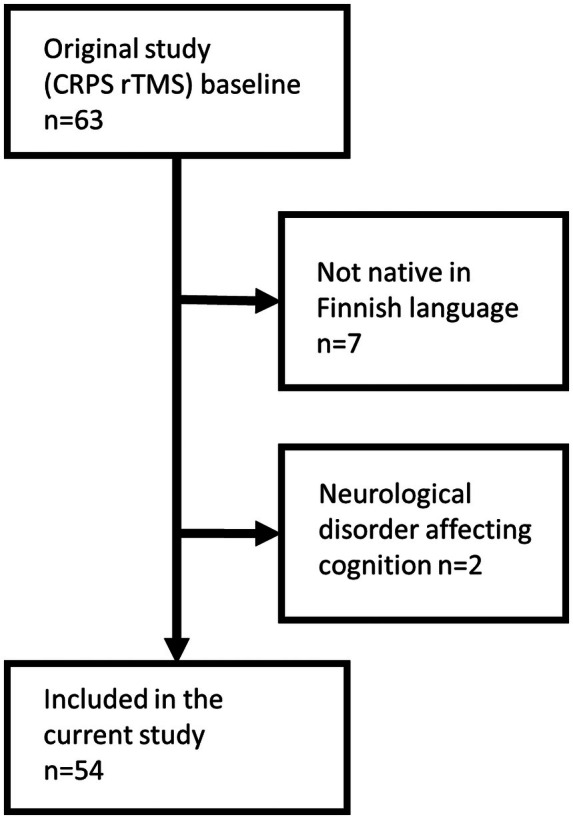
Flow chart of the study.

The total number of patients recruited for the CRPS rTMS study was 63. Two patients were diagnosed with a neurological disorder that could affect cognitive function (one traumatic brain injury, one Parkinson's disease); seven patients were not fluent in the Finnish language. These patients were all excluded from this study, giving a final number of 54 patients included in this study (flow chart, Figure 1).

Descriptive statistics for the patients are presented in Table 3. Medical co‐morbidities reported were migraine [12 (22%)], asthma [13 (24%)], hypertension [10 (19%)], hypothyroidism [9 (17%)], hypercholesterolemia [5 (9%)], rheumatoid arthritis [3 (6%)], diabetes (type II) [4 (7%)], hearing loss [3 (6%)], reflux disease [2 (4%)], cardiac arrhythmia [3 (6%)] and hypogammaglobulinemia [2 (4%)]. Medications potentially affecting cognitive function include anticonvulsants, antidepressants, corticosteroids, opioids (strong opioids were an exclusion criterion), muscle relaxants, and benzodiazepines. Of these, 16 patients were taking one medication, 23 patients two, 7 patients three, 5 patients four, with one patient taking five different medications.

**TABLE 3:** Descriptive statistics of patients at the baseline examination.

*N*	54
Age [mean (SD)]	42.3 (13.5)
Women [*n* (%)]	47 (87)
Education [*n* (%)]	
Low	8 (15)
Medium	14 (27)
High	28 (54)
Extremity affected (*n* right/*n* left)	30/24
CRPS on dominant/non‐dominant hand	34/20
Duration of pain [*n* (%)]	
6 months–1 year	12 (22.6)
1–3 years	22 (41.5)
> 3 years	19 (35.8)
CRPS type (*n* 1/*n* 2)	41/13
CRPS severity [mean (SD)]	12.2 (1.7)
Medication [*n* (%)]^a^	
Anticonvulsants	37 (68.5)
Antidepressants	27 (50.0)
Corticosteroids	1 (1.9)
Mild opioids	21 (38.9)
Muscle relaxants	5 (9.3)
Benzodiazepines	5 (9.3)
Pain intensity [mean (SD)]	5.9 (1.3)
Pain interference—activity [mean (SD)]	5.8 (1.7)
Pain interference—affective [mean (SD)]	6.0 (2.0)
Pain NRS [mean (SD)]	7.9 (1.2)
Disability [mean (SD)]	89.4 (17.6)
Insomnia symptoms [mean (SD)]	13.6 (6.4)
Depressive symptoms [mean (SD)]	14.8 (7.9)
State Anxiety [mean (SD)]	38.4 (10.6)
Pain catastrophizing [mean (SD)]	18.2 (10.1)
Psychological resilience [mean (SD)]	76.0 (13.2)

*Note:* Age (years); Education (low = primary school; medium = secondary school high = university of applied sciences or higher); CRPS severity (CRPS Severity Scale [0 to 16]).

Abbreviations: SD, standard deviation; *n*, number.

^a^ Only medications potentially affecting cognition reported here; pain severity and interference (Brief Pain Inventory [0 to 10]); pain NRS (worst pain on numerical rating scale [0 to 10]); Disability (disabilities of the arm, shoulder, and hand questionnaire [0 to 100]); Insomnia symptoms (Insomnia Severity Index [0 to 28]); Depressive symptoms (Beck Depression Inventory‐II, [0 to 63]); State anxiety (State Trait Anxiety Inventory [20 to 80]); Pain catastrophizing (Pain Catastrophizing Scale [0 to 52]); Psychological resilience (Resilience Scale‐14 [14 to 98]).

Duration of pain was reported on an ordinal scale (less than a year, 1–2, 2–3, 3–4, 4–5 and over 5 years) and categorised into three groups: < 1, 1–3, > 3 years. Level of education was recorded as low (primary school only), medium (secondary or vocational school), or high (university of applied sciences or higher). Eighteen patients reported working full or part time (33%), 26 (48%) retired, on sick leave or on a temporary disability pension; two patients (4%) were on maternity leave and eight (15%) were studying. Raw scores for the neuropsychological tests are presented in Table 4.

**TABLE 4:** Raw scores of neuropsychological tests.

Test (selected variable)	*n*	Mean	SD
Reasoning			
Block design (WAIS‐IV)	54	41.69	11.96
Similarities (WAIS‐IV)	53	25.75	3.81
Memory			
Word list (WMS‐III)	53	30.98	5.82
Word list delayed recall (WMS‐III)	53	6.70	2.42
Digit span forward (WMS‐III)	53	8.19	1.43
Digit span backward (WMS‐III)	53	8.11	2.13
Attention			
TMTB	54	83.02	46.17
TMTB‐TMTA	54	51.28	40.43
Executive function			
Verbal fluency phonemic	54	17.39	5.31
Drawing fluency	53	5.57	2.49
Brixton	54	270.56	93.18
Brixton error	54	15.35	9.51
B–W dual task decrement	53	39.85	17.87
Processing speed			
TMTA	54	31.47	12.10
Mental rotation			
Manikin	54	49.33	19.85

Abbreviations: B–W Bourdon–Wiersma dot cancellation test; TMT, Trail Making Test; WAIS‐IV, Wechsler Adult Intelligence Scale, 4th edition; WMS‐III, Wechsler Memory Scale, 3rd edition.

### 3.2 Subjective Cognitive Difficulties

In the neuropsychological examination, 44 patients (86%) reported that CRPS had affected their cognitive abilities; seven patients (14%) reported no subjective impact, while for 3 patients this information was missing. Patients gave an average score of 6.1 (SD 2.1) on a 0 to 10 (0 very bad; 10 very good) scale. Patients who had reported that CRPS had affected their cognitive ability (*n* = 42) reported significantly lower rates for their subjectively judged cognitive abilities in the interview (5.6 [1.9]), compared with patients (*n* = 6) who had not experienced any effect on their cognition (9.2 [0.4]), (*p* ≤ 0.001). In the regression analysis, concurrent depressive symptoms predicted subjective cognitive ability (*B* = −0.16, *p* ≤ 0.001), when the effect of age was controlled.

Patients' subjective judgements of their cognitive functioning were not associated with objective performance on the neuropsychological tests used in this study (Table 5). In group comparisons, there was no difference in cognitive performance in separate neuropsychological tests after Bonferroni corrections between patients who reported or did not report cognitive decline. As a continuous variable, subjective judgements of cognitive functioning were not associated with neurocognitive functioning in multivariate regression analysis (Wilk's ᴧ = 0.69, *F* = 1.01, *p* = 0.47).

**TABLE 5:** Frequencies of objective vs. subjective cognitive impairment.

		Objective cognitive decline		
		Yes	No	Total
Subjective cognitive decline	Yes	14 (93%)	30 (83%)	44 (86%)
	No	1 (7%)	6 (17%)	7 (14%)
	Total	15 (100%)	36 (100%)	51 (100%)

*Note:* Objective decline: performance below clinical cutoff point on three different tests. Subjective decline: Positive answer to question ‘Do you feel that CRPS has caused you problems with memory and information processing?’.

Patients with subjective cognitive difficulties (*n* = 44) scored higher for depressive symptoms than those without (*n* = 7) ([15.91 (7.19) vs. 5.00 (2.94), *p* ≤ 0.001], Affective Pain Interference [6.28 (1.74) vs. 3.39 (1.76), *p* ≤ 0.001], Insomnia Symptoms [14.66 (5.75) vs. 5.14 (4.53), *p* ≤ 0.001], Disability [91.37 (16.72) vs. 72.43 (14.00), *p* ≤ 0.001], and anxiety [39.26 (8.69) vs. 27.49 (9.92), *p* = 0.019]). Patients with subjective cognitive difficulties, compared with those without, had lower scores for resilience (74.70 [13.14] 86.57 [5.86], *p* ≤ 0.001). After Bonferroni corrections (*α* = 0.005) these comparisons remained significant except for anxiety. There was no difference in the level of Pain Catastrophizing, Activity Pain Interference, Pain Intensity, and number of CRPS symptoms (CRPS severity scale, CSS) among patients with or without subjective cognitive difficulties. CRPS type (1 vs. 2) was not associated with either the presence of subjective cognitive difficulties (*p* = ns) or with the subjectively judged cognitive ability (*p* = ns).

### 3.3 Cognitive Performance Compared With the Healthy Population

On average, when calculating the means of age‐controlled performance, the patients performed within the normal range of cognitive function.

In this study, 16 patients (30%) performed below age‐corrected clinical cutoff points on three or more tests and were classified as having cognitive decline. After Bonferroni correction (*α* = 0.005) these groups did not differ on disability (*p* = 0.48), depressive symptoms (*p* = 0.15), pain intensity (*p* = 0.08), Affective Pain Interference (*p* = 0.36), Activity Pain Interference (*p* = 0.34), insomnia symptoms (*p* = 0.04), anxiety (*p* = 0.32), psychological resilience (*p* = 0.40), CRPS severity (*p* = 0.35), or pain catastrophizing (*p* = 0.12). Number of different medications taken did not differ between patients with or without cognitive decline (*p* = 0.29).

On the Bourdon–Wiersma test, there was no difference in attentional processing (number of errors) between left (median 2, IQR [0.5–4.5]) and right (median 2, IQR [1–4]) (*p* = 0.91) sides for the visual stimulus task.

### 3.4 Cognitive Performance and CRPS‐Related Factors

Fifteen neuropsychological tests that were normally distributed were used as dependent variables in separate linear regression models and binary logistic regression models (for CRPS type), controlled for age, medication, depressive symptoms, and insomnia symptoms. CSS, Pain Intensity, Pain Interference, and either Affective or Activity Pain Interference did not predict neuropsychological test performances (Table 6). CRPS type (1 or 2) was not associated with any cognitive test performance after Bonferroni corrections (*p* = ns).

**TABLE 6:** Separate linear regression models for different dependent variables. All models controlled for age, medication, depressive symptoms, and insomnia symptoms; *p*‐values for each dependent variable are presented in the table.

Independent variables	Dependent variables														
	Reasoning		Memory				Attention		Executive function					Processing speed	Mental rotation
	Block design (WAIS‐IV)	Similarities (WAIS‐IV)	Word list (WMS‐III)	Word list Delayed Recall (WMS‐III)	Digit span forward (WMS‐III)	Digit Span backward (WMS‐III)	TMTB	TMTB‐TMTA	Verbal fluency phonemic	Drawing fluency	Brixton	Brixton errors	B–W dual task decrement	TMTA	Manikin
	*p*	*p*	*p*	*p*	*p*	*p*	*p*	*p*	*p*	*p*	*p*	*p*	*p*	*p*	*p*
	*B*	*B*	*B*	*B*	*B*	*B*	*B*	*B*	*B*	*B*	*B*	*B*	*B*	*B*	*B*
	CI	CI	CI	CI	CI	CI	CI	CI	CI	CI	CI	CI	CI	CI	CI
CRPS symptoms	0.48	0.23	0.62	0.73	0.76	0.89	0.23	0.38	0.78	0.03	0.58	0.34	0.05	0.15	0.90
	0.61	−0.4	−0.23	0.06	0.04	−0.03	−4.11	−2.76	0.13	0.46	−4.27	−0.67	−2.81	−1.35	−0.21
	[−1.1, 2.33]	[−1.06, 0.27]	[−1.13, 0.68]	[−0.29, 0.41]	[−0.21, 0.29]	[0.39, 0.34]	[−10.91, 2.67]	[−9.00, 3.49]	[−0.80, 1.06]	[0.05, 0.87]	[−19.49, 10.95]	[−2.07, 0.73]	[−5.62, 0.003]	[−3.21, 0.51]	[−3.33, 2.92]
Pain Intensity	0.49	0.25	0.40	0.30	0.41	0.33	0.07	0.08	0.90	0.30	0.23	0.42	0.51	0.47	0.28
	0.83	−0.54	−0.54	−0.26	0.14	0.25	−8.82	−7.85	−0.08	0.31	−12.89	0.80	−1.37	−0.98	2.37
	[−1.58, 3.24]	[−1.47, 0.40]	[−1.82, 0.74]	[−0.75, 0.24]	[−0.21, 0.50]	[−0.26, 0.76]	[−18.42, 0.78]	[−16.53, 0.84]	[−1.38, 1.22]	[−0.29, 0.91]	[−33.98, 8.20]	[−1.17, 2.76]	[−5.47, 2.74]	[−3.68, 1.73]	[−1.97, 6.71]
Pain interference—affective	0.38	0.16	0.62	0.34	0.01	0.10	0.10	0.15	0.87	0.30	0.95	0.05	0.55	0.20	0.61
	0.87	0.56	0.26	−0.20	0.38	0.35	−6.70	−5.28	0.09	−0.26	−0.57	1.55	1.02	−1.42	0.92
	[−1.11, 2.85]	[−0.22, 1.33]	[−0.81, 1.34]	[−0.61, 0.21]	[0.11, 0.65]	[−0.07, 0.76]	[14.63, 1.24]	[−12.50, 1.95]	[−0.98, 1.16]	[−0.77, 0.24]	[−18.19, 17.05]	[−0.01, 3.11]	[−2.36, 4.39]	[−3.62, 0.78]	[−2.69, 4.52]
Pain interference—active	0.24	0.84	0.50	0.20	0.10	0.08	0.01	0.03	0.63	0.42	0.15	0.01	0.49	0.07	0.41
	1.09	0.08	−0.33	−0.24	0.22	0.34	−9.20	−7.35	−0.24	0.19	−11.69	1.85	−1.10	−1.85	1.38
	[−0.75, 2.92]	[−0.65, 0.80]	[−1.32, 0.65]	[−0.62, 0.13]	[−0.05, 0.48]	[−0.04, 0.72]	[−16.35, −2.05]	[−13.92, −0.79]	[−1.24, 0.76]	[−0.28, 0.65]	[−27.80, 4.42]	[0.43, 3.27]	[−4.26, 2.05]	[−3.87, 0.17]	[−1.97, 4.74]
Pain catastrophizing	0.14	0.37	0.04	0.08	0.01	0.54	0.02	0.14	0.67	0.19	0.53	0.69	0.27	< 0.001*	0.77
	−0.25	−0.06	−0.18	−0.06	−0.07	−0.02	1.57	0.91	−0.04	−0.06	−0.09	0.06	0.32	**0.66**	−0.09
	[−0.58, 0.08]	[−0.19, 0.08]	[−0.35, −0.01]	[−0.13, 0.01]	[−0.11, −0.02]	[−0.09, 0.05]	[0.26, 2.87]	[−0.31, 2.14]	[−0.22, 0.14]	[−0.14, 0.03]	[−3.78, 1.97]	[−0.22, 0.33]	[−0.25, 0.88]	**[0.34, 0.98]**	[−0.70, 0.52]
Psychological resilience	0.63	0.46	0.64	0.93	0.81	0.37	0.96	0.63	0.03	0.17	0.03	0.67	0.68	0.16	0.11
	−0.07	−0.04	−0.03	0.002	0.01	0.03	0.03	0.24	−0.15	−0.05	2.56	0.05	−0.10	−0.21	0.39
	[−0.33, 0.20]	[−0.15, 0.07]	[−0.18, 0.11]	[−0.05, 0.06]	[−0.03, 0.04]	[−0.03, 0.08]	[−1.07, 1.13]	[−0.75, 1.23]	[−0.28, −0.01]	[−1.11, 0.02]	[0.31, 4.80]	[−0.17, 0.26]	[−0.55, 0.36]	[−0.50, 0.09]	[−0.09, 0.86]

*Note:* Since 15 different tests were run due to 15 neuropsychological test parametres. Bonferroni corrections were made and the significance level for *p*‐value was set to 0.003. Grey colour represents non‐significant results, black represents marginal *p*‐value (0.003–0.05) and bold represents significant results. All models were controlled for age, medication, depressive symptoms, and insomnia symptoms. Pain severity and interference (Brief Pain Inventory), pain catastrophizing (Pain Catastrophizing Scale), psychological resilience (Resilience Scale‐14). *p < 0.001. Bolded coefficients indicate statistically significant predictors.

Abbreviations: B–W, Bourdon–Wiersma test; TMTA, Trail Making Test A; TMTB, Trail Making Test B; WAIS‐IV, Wechsler Adult Intelligence Index–IV; WMS‐III, Wechsler Memory Scale–III.

### 3.5 Cognitive Performance, Psychological Resilience, and Pain Catastrophizing

In the linear regression model, pain catastrophising was associated with performance in TMTA, when controlled for age, medication, depressive, and insomnia symptoms (Table 6). The association between psychological resilience and neuropsychological test performance was not statistically significant after Bonferroni corrections.

## 4 Discussion

### 4.1 Main Results

The main result of this study is that a subgroup, comprising 30% of the CRPS patients, shows cognitive decline. However, at the group level (all patients), only verbal fluency (indicative of executive function) fell below the cutoff point of clinically normal performance. Pain catastrophising was associated with poorer performance in the TMTA, a measure of processing speed, even after controlling for age, medication, insomnia, and depressive symptoms. Type of CRPS (1 or 2), pain intensity, pain interference, CRPS symptom severity or psychological resilience was not associated with objectively measured cognitive function in this study. Subjective cognitive difficulties and CRPS type were not associated with objective cognitive performance but were associated with affective pain interference, disability, anxiety, depressive and insomnia symptoms.

### 4.2 Comparison With Previous Studies

In this study, 70% of the patients did not demonstrate neurocognitive impairment, a larger proportion than the 35% reported in a previous study (Libon et al. 2010). That study identified mild executive deficits in 42% of patients and global cognitive impairment in 22% (Libon et al. 2010). Even though direct comparison of the studies is not possible due to different methodologies, it can be mentioned that similar groups were not present in our study. Consistent with earlier research (Libon et al. 2010; Reinersmann et al. 2010), verbal fluency was impaired while working memory remained intact. Lack of association between neuropsychological functioning and the number of CRPS symptoms or illness duration is also in line with previous work (Libon et al. 2010). Further, our finding that executive function might be affected by CRPS agrees with the previous finding of mild executive deficits in a subgroup of patients (Libon et al. 2010). The effect of CRPS type, 1 or 2, has not previously been studied in relation to cognitive decline, but our results show that the diagnostic category does not influence cognitive performance, either subjectively or in objective tests.

We found no differences between left and right sides for visual attention in a traditional pen‐and‐paper test. This is in line with previous research where differences in attentional sides were not seen in CRPS patients in visual neglect tests traditionally used in post‐stroke patients with hemispatial neglect (Kolb et al. 2012). Published research is reasonably consistent in agreeing that the hemispatial neglect framework, validated for post‐stroke brain lesions, is not sufficient to assess cognitive functions in CRPS (Halicka et al. 2020).

Pain catastrophizing has been reported to be correlated with lower structural integrity in the prefrontal cortex in CRPS patients, suggesting that pain catastrophizing may be associated with dysfunction of the prefrontal white matter (Im et al. 2021). Frontal areas are known to be involved in executive functions (Jones and Graff‐Radford 2021). In the executive function test of our study, CRPS patients performed worse than expected for the healthy population, and there was a tendency towards an association between executive function and pain interference, pain catastrophizing, and psychological resilience. However, these possible associations were not significant.

The level of pain catastrophizing in the current study was lower than previously found in CRPS patients (18.2 [10.1] vs. 34.8 [14.4], *p* < 0.001) (Im et al. 2021). Cultural differences between Korean and Finnish patients might explain at least part of the discrepancy. In another recent study (Ysidron et al. 2021), pain catastrophizing moderated the effect of pain resilience on attention‐demanding cognitive task performance during experimental pain, but neither pain resilience nor pain catastrophizing alone was associated with cognitive task performance. The effects of CRPS on cognitive functioning are most likely multimodal. Due to cross‐sectional design we do not have information about the pre‐CRPS cognitive capacity and the individuals' magnitude of cognitive decline. Future research should explore the effect of pain catastrophizing and resilience on executive function in more depth.

In our study, the level of resilience was similar to that reported in other Finnish studies in both healthy individuals (76.3 [10.7]) (Losoi 2013) and also in patients with somatic health problems (80.0 (10.9)) (Losoi et al. 2015) (77.7 [12.9]) (Liesto et al. 2020). Previous studies of resilience in CRPS patients used different resilience measurements than ours (Schrier et al. 2019; Wertli et al. 2023). The authors found the level of resilience to be above the population average but suggested that this might be due to selection bias (Schrier et al. 2019). This study is the first to examine resilience and neurocognitive function in CRPS patients and showed no associations between them.

For our patients, the education level was 15% primary school only, in 27% secondary school, and in 54% university of applied science or higher. In the Finnish population aged 20–79 years, the proportions are 18%, 45% and 37% respectively (Statistics Finland). Thus, we had a greater proportion of highly educated patients (*p* = 0.017) than would have been expected. The overrepresentation of patients with better cognitive reserve might have affected the present results as the patients might have been more resistant to the neurocognitive deficits associated with CRPS, found in higher percentages in previous studies (Libon et al. 2010).

Subjective and objective cognitive performance were not correlated in our study. This is a common finding in previous neuropsychological studies with different conditions (Hutchinson et al. 2012; Siciliano et al. 2021). In our study, subjective cognitive difficulties were related to affective pain interference, disability, anxiety, and depressive or insomnia symptoms.

Medication, especially opioids, can impair attention (Allegri et al. 2019) but untreated pain also impairs cognition (Jang et al. 2022). No medication effects on cognition were identified in this study.

### 4.3 Clinical Significance

CRPS is a multifactorial pain syndrome and best addressed within the biopsychosocial framework (Melf‐Marzi et al. 2022). Clinical guidelines emphasise physiotherapy and occupational therapy as key rehabilitation components (Harden et al. 2022; Melf‐Marzi et al. 2022). Furthermore, integrating neurocognitive or neuropsychobehavioural approaches or strategies may enhance sensorimotor integration (Melf‐Marzi et al. 2022).

Neurocognitive research in CRPS enhances understanding of its mechanisms and treatments. Neuropsychological rehabilitation alleviates cognitive symptoms and enhances functioning and quality of life through skills practice, psychoeducation, and compensatory strategies. In CRPS, cognitive and clinical symptoms may correlate (Halicka et al. 2020), although in this study these associations could not be confirmed in the main. Pain severity and other CRPS symptoms have been shown to be associated with the extent of functional reorganisation of primary sensory and motor cortices (Pleger et al. 2005). Reversing this reorganisation during CRPS treatment has been associated with symptom improvement (Pleger et al. 2005). Consequently, neuropsychological rehabilitation of cognitive impairments in CRPS may alleviate pain and other symptoms due to brain plasticity accountable for both effects. In this respect, the current findings are interesting as more severe sensorimotor handicap induced by CRPS was associated with better neuropsychological coping. Furthermore, CRPS patients with cognitive deficits formed a subgroup, suggesting that rehabilitation should be tailored based on the particular neuropsychological profile and sensorimotor deficits. Including neuropsychological assessment and rehabilitation within a multidisciplinary care could be particularly beneficial for CRPS patients with cognitive symptoms, although research into this aspect is scarce (Halicka et al. 2020).

The modern work environment is increasingly demanding cognitively, underscoring the importance of structured, multidisciplinary assessment that also encompasses neuropsychological evaluation. Implementing standardised evaluations for all CRPS patients would facilitate better management of their rehabilitation, as well as assessment of health insurance benefits.

### 4.4 Strengths and Limitations

A strength of the study is the relatively large cohort for such a rare disease. However, statistically, the sample size is still small, perhaps rendering the results prone to type II errors. Another strength is the use of a comprehensive battery of neuropsychological tests, where previous studies have used relatively few. Our study also adds important knowledge about psychological aspects, such as pain catastrophising and resilience, in association with neurocognitive function.

A limitation is that the cross‐sectional study design does not allow assessment of pre‐CRPS cognitive ability. Chronic pain is associated with cognitive dysfunctions, particularly of memory, attention, executive function, and processing speed (Khera and Rangasamy 2021). This raises the question of which dysfunctions are specific to CRPS and which are related to chronic pain in general. Future studies should include control groups with other pain conditions or without pain. Another limitation is that we do not have recorded details on how the CRPS motor dysfunction affected the performance in some of the neuropsychological tests (e.g., if the test was performed with non‐dominant hand).

## 5 Conclusion

We identified a substantial subgroup of CRPS patients with cognitive decline. Executive function seemed to be affected, and pain catastrophising was associated with slower processing speed. Different types of tailored interventions based on neuropsychological profiling, in addition to those based on traditional clinical symptoms and signs of CRPS, should be applied in future rehabilitation programmes. For these, it would be important to conduct prospective studies to help us understand why some patients develop cognitive symptoms while others do not.

## Conflicts of Interest

The authors declare no conflicts of interest.
